# *In Silico* Analysis and Experimental Validation of Active Compounds from *Cichorium intybus* L. Ameliorating Liver Injury

**DOI:** 10.3390/ijms160922190

**Published:** 2015-09-14

**Authors:** Guo-Yu Li, Ya-Xin Zheng, Fu-Zhou Sun, Jian Huang, Meng-Meng Lou, Jing-Kai Gu, Jin-Hui Wang

**Affiliations:** 1 Research Center for Drug Metabolism, College of Life Science, Jilin University, Changchun 130012, China; E-Mail: gyl197908@163.com; 2 School of Traditional Chinese Materia Medica, Shenyang Pharmaceutical University, Shenyang 110016, China; E-Mails: zhengyaxinxyz@163.com (Y.-X.Z.); sunfuzhou1989@126.com (F.-Z.S.); profhj@163.com (J.H.); 3 School of Pharmacy, Shihezi University, Shihezi 832002, China; E-Mail: lmm198508@163.com

**Keywords:** *Cichorium intybus* L., *in silico* analysis, acute liver injury, Akt-1, caspase-1

## Abstract

This study aimed at investigating the possible mechanisms of hepatic protective activity of *Cichorium intybus* L. (chicory) in acute liver injury. Pathological observation, reactive oxygen species (ROS) detection and measurements of biochemical indexes on mouse models proved hepatic protective effect of *Cichorium intybus* L. Identification of active compounds in *Cichorium intybus* L. was executed through several methods including ultra performance liquid chromatography/time of flight mass spectrometry (UPLC-TOF-MS). Similarity ensemble approach (SEA) docking, molecular modeling, molecular docking, and molecular dynamics (MD) simulation were applied in this study to explore possible mechanisms of the hepato-protective potential of *Cichorium intybus* L. We then analyzed the chemical composition of *Cichorium intybus* L., and found their key targets. Furthermore, *in vitro* cytological examination and western blot were used for validating the efficacy of the selected compounds. *In silico* analysis and western blot together demonstrated that selected compound **10** in *Cichorium intybus* L. targeted Akt-1 in hepatocytes. Besides, compound **13** targeted both caspase-1 and Akt-1. These small compounds may ameliorate liver injury by acting on their targets, which are related to apoptosis or autophagy. The conclusions above may shed light on the complex molecular mechanisms of *Cichorium intybus* L. acting on hepatocytes and ameliorating liver injury.

## 1. Introduction

*Cichorium intybus* L. (also known as chicory) is a perennial herb from the *Asteraceae* family with both alimentary use and medicinal use. It is 40–100 cm in height, usually with bright blue flowers, rarely white or pink. It has been reported that fresh chicory typically contains 68% inulin, 14% sucrose, 5% cellulose, 6% protein, 4% ash, and 3% other compounds, while dried chicory contains approximately 98% inulin and 2% other compounds [1]. *Cichorium intybus* L. is one of the most promising novel candidates among the carbohydrates with potential for both food and non-food utilization. With the function of diuresis, promoting digestion, curing jaundice, and benefiting kidney and liver function, according to the traditional herbalist doctor, it has been implemented in folk medicine from North Africa to South Asia for several centuries. It is worth noting that *Cichorium intybus* L. possesses a wide range of pharmacological properties according to the literature, including hepato-protective, anti-inflammatory, anti-malarial, anti-diabetic effects, *etc.* [2,3,4,5,6]. In the present study, *Cichorium intybus* L. extract (CIE) exhibited a hepato-protective effect, as demonstrated by a significant decrease in aspartate aminotransferase (AST) and alanine aminotransferase (ALT) concentrations and the prevention of histopathological changes in the liver of mice with CCl_4_-induced hepatotoxicity. Moreover, CIE attenuated the reduction of glutathione (GSH) and superoxide dismutase (SOD), and decreased malondialdehyde (MDA) levels in mice with CCl_4_-induced hepatotoxicity [2,7].

Although *Cichorium intybus* L. has distinct hepatic disease protection effects, its pharmacodynamic material basis and molecular mechanisms still need far more research. In this study, we analyzed the chemical composition of *Cichorium intybus* L., then certificated the structure of 10 compounds and determined their potential target proteins via SEA (similarity ensemble approach) docking. Based on the docking results, we chose two compounds (compounds **10** and **13**) and two of their key target proteins (Akt-1 and caspase-1) to conduct further study after applying gene ontology (GO) and KEGG orthology (KO) to find their functions and connections. Additionally, molecular modeling, molecular docking and MD simulation results proved good interaction between two small compounds and their targets. Finally, experimental validation, including western blot, was conducted to verify the active substance basis of the hepatic disease protection of *Cichorium intybus* L. *In silico* analysis and experimental validation together demonstrated that selected compound **13** in *Cichorium intybus* L. targeted RAC-α serine/threonine-protein kinase (Akt-1) and caspase-1 in hepatocytes, while compound 10 targeted Akt-1. What has been discussed above indicates that these two compounds in *Cichorium intybus* L. may ameliorate liver injury.

## 2. Results

### 2.1. The Effect of Cichorium intybus L. on Liver Injury Protection

**Figure 1:**
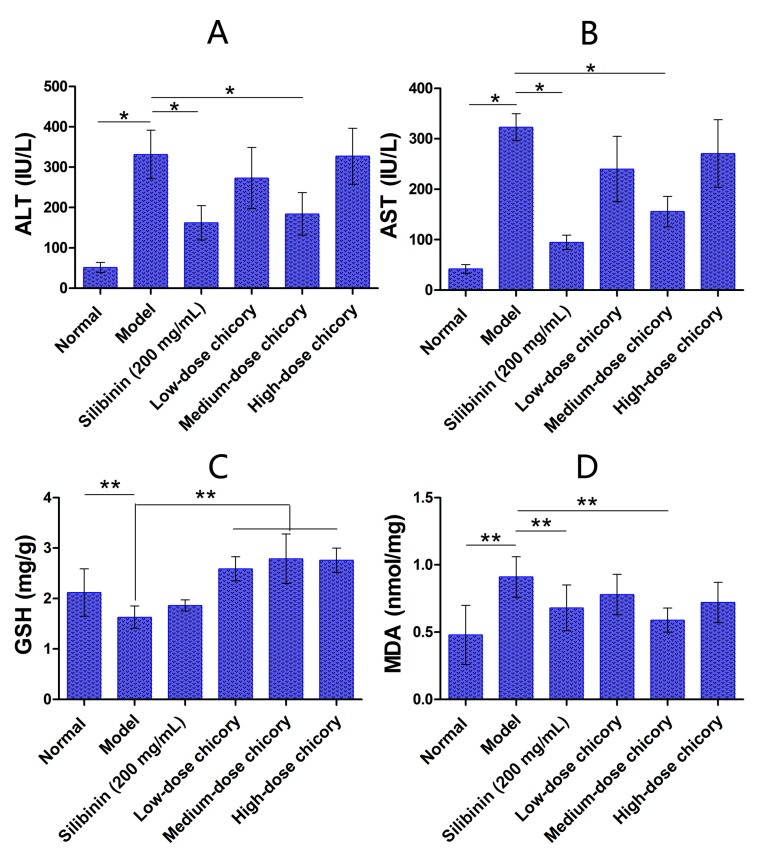
(**A**) Serum ALT (alanine aminotransferase) level in mice; (**B**) serum AST (aspartate aminotransferase) level in mice; (**C**) liver tissue GSH (glutathione) level in mice; and (**D**) liver tissue MDA (malondialdehyde) level in mice. *, *p* < 0.05; **, *p* < 0.01.

Compared with the injury model group, serum ALT level and AST level in the middle-dose group (6.0 g/kg chicory) were significantly reduced, while there were no obvious changes in the low-dose group (2.0 g/kg chicory) and the high-dose group (18.0 g/kg chicory) (Figure 1A,B).

Liver tissue GSH level (Figure 1C) was significantly reduced and MDA level (Figure 1D) went up in the middle-dose group, while there were no obvious changes in low- and high-dose groups, compared with the model group.

**Figure 2:**
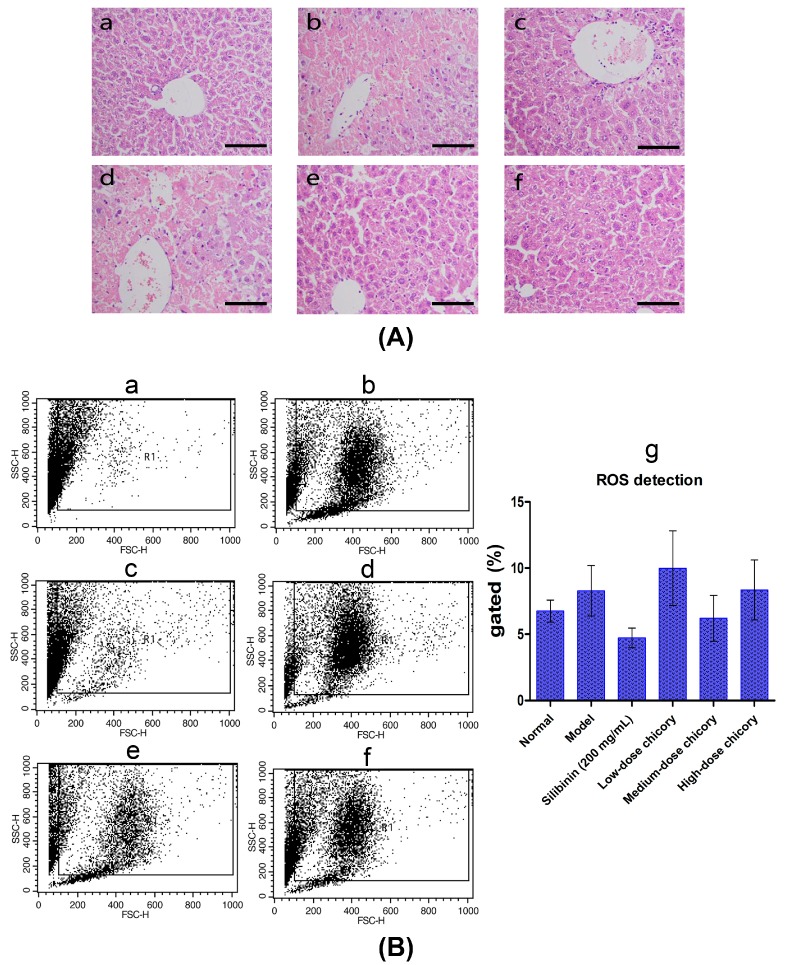
(**A**) Liver tissue histopathological observation results, (**a**) normal control; (**b**) liver injury model; (**c**) positive drug silibinin control (200 mg/mL); (**d**) low-dose drug group (2.0 g/kg chicory); (**e**) middle-dose group (6.0 g/kg chicory); and (**f**) high-dose group (18.0 g/kg chicory); and scale bar = 100 μm; (**B**) ROS detection result, (**a**) normal control; (**b**) liver injury model; (**c**) positive drug silibinin control (200 mg/mL); (**d**) low-dose drug group (2.0 g/kg chicory); (**e**) middle-dose group (6.0 g/kg chicory); (**f**) high-dose group (18.0 g/kg chicory); and (**g**) quantitative analysis of ROS detection. FSC-H and SSC-H represent cell numbers and fluorescence intensity, respectively.

Figure 2A–F shows the liver tissue histopathological observation results of all six groups. It can been seen from the figure that liver cells were arranged radially around the central veios in the normal control, while acute pathological changes occurred in liver injury model group, including centrilobular hepatic necrosis, the deformation of macrophage and ballooning, and a large number of inflammatory cells infiltration. The silibinin group and middle-dose group can ameliorate the injury apparently.

The result of ROS (reactive oxygen species) detection further demonstrated the protective activity of *Cichorium intybus* L. Compared with the normal group, many more cells from the model group died. The middle-dose of *Cichorium intybus* L. could obviously protect hepatocytes from apoptosis, the same effect as Silibinin, while the low-dose group or high-dose group did not show the same effect (Figure 2B).

### 2.2. Small Molecule Compounds and Target Proteins

We identified structures of 10 small molecule compounds in *Cichorium intybus* L., which are shown in Table 1. Among them, compounds **2** and **5** were new compounds that had not been reported; compound **6** was isolated from the genus for the first time; and compound **10** was isolated from this plant for the first time. We then found the target proteins of eight of them (Table 2). After we found the functions of these proteins, we chose compound **10** (methyl 4-hydroxyphenylacetate) and compound **13** (4-hydroxylphenylacetic acid) and their target proteins to conduct our further study.

**Table 1:** The structures of compounds in *Cichorium intybus* L.

Number	Name	Structure	Identification Means
**2**	Intyboate B	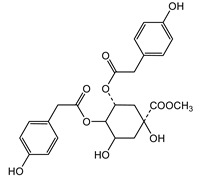	^13^C-NMR; ^1^H-NMR; HMBC; HSQC; NOESY; m.p.; UV; CD; HR-ESI-TOF-MS
**5**	3a*R*-santamarine	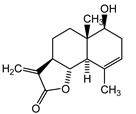	^13^C-NMR; ^1^H-NMR; HMBC; HMQC; NOSEY; m.p.; UV; CD; HR-ESI-TOF-MS
**6**	Aurantiamide acetate	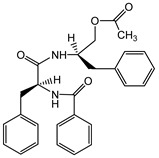	^13^C-NMR; ^1^H-NMR; HMBC; HMQC; m.p.; UV; CD; HR-ESI-TOF-MS
**7**	Luteolin-7-*O*-β-d-glucoside	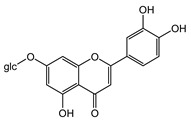	^13^C-NMR; ^1^H-NMR; m.p.; HR-ESI-TOF-MS
**8**	Luteolin	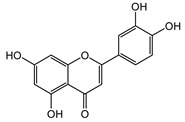	^13^C-NMR; ^1^H-NMR; HR-ESI-TOF-MS
**9**	Caffeic acid	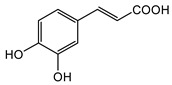	^13^C-NMR; ^1^H-NMR; HR-ESI-TOF-MS
**10**	Methyl 4-hydroxyphenylacetate	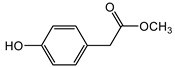	^13^C-NMR; ^1^H-NMR; HMBC; HMQC; HR-ESI-TOF-MS
**11**	β-sitosterol	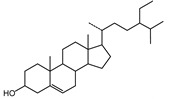	TLC
**12**	Daucosterol	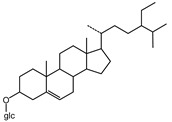	TLC
**13**	4-Hydroxylphenylacetic acid	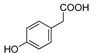	HR-ESI-TOF-MS

HMBC, heteronuclear multiple-bond correlation; HSQC, heteronuclear singular quantum correlation; NOESY, nuclear overhauser effect spectroscopy; m.p., melting point; UV, ultraviolet spectrum; CD, circular dichroism spectrum; HR-ESI-TOF-MS, high-resolusion electrospray-ionization time-of-flight mass spectrometry; TLC, thin-layer chromatography.

**Table 2:** Target proteins of small compounds in *Cichorium intybus* L.

Number	Name	ZINC	Target	UniProtKB AC	Recommended Name
**1**	Intyboate A	None	ACE_MOUSE_100	P09470	Angiotensin-converting enzyme
**2**	Intyboate B	None	ACE_MOUSE_100	P09470	Angiotensin-converting enzyme
**5**	3a*R*-santamarine	ZINC05663288	None	None	None
**7**	Luteolin-7-*O*-â-d-glucoside	ZINC03947434	TOP1_MOUSE_10000	Q04750	DNA topoisomerase 1; Interleukin-5; Aldo-ketoreductase family 1 member C21; Multidrug resistance protein 3; γ-Aminobutyric acid receptor subunit α-1
			IL5_MOUSE_10000	P04401	
			AK1CL_MOUSE_10000	Q91WR5	
			MDR3_MOUSE_10000	P21440	
			GBRA1_MOUSE_10	P62812	
**8**	Luteolin	ZINC18185774	AK1CL_MOUSE_10000	Q91WR5	Aldo-ketoreductase family 1 member C21; Multidrug resistance protein 3; Tyrosinase; γ-Aminobutyric acid receptor subunit α-1; Interleukin-5; Phosphatidylinositol 4,5-bisphosphate 3-kinase catalytic subunit α isoform; Dual specificity protein phosphatase 1; Solute carrier family 22 member 6
			MDR3_MOUSE_100	P21440	
			TYRO_MOUSE_10000	P11344	
			GBRA1_MOUSE_1000	P62812	
			IL5_MOUSE_10000	P04401	
			PK3CA_MOUSE_10000	P42337	
			DUS1_MOUSE_10000	P28563	
			S22A6_MOUSE_10000	Q8VC69	
**9**	Caffeic acid	ZINC00058172	MDR2_MOUSE_10000	None	Multidrug resistance protein 1B; Multidrug resistance protein 3; Transcription factor HES-1; Dual specificity tyrosine-phosphorylation-regulated kinase 1A; Epidermal growth factor receptor; Carbonic anhydrase 15; Retinoic acid receptor RXR-α
			MDR1_MOUSE_10000	P06795	
			MDR3_MOUSE_10000	P21440	
			HES1_MOUSE_10000	P35428	
			DYR1A_MOUSE_10000	Q61214	
			EGFR_MOUSE_10000	Q01279	
			CAH15_MOUSE_10000	Q99N23	
			RXRA_MOUSE_1000	P28700	
**10**	Methyl 4-hydroxyphenylacetate	ZINC00395674	ACE_MOUSE_1000	P09470	Angiotensin-converting enzyme; Solute carrier family 22 member 20; Solute carrier family 22 member 6; Platelet-derived growth factor receptor α (PDGFRα); Carbonic anhydrase 15; Mu-type opioid receptor; Bifunctional epoxide hydrolase 2; RAC-α serine/threonine-protein kinase (Akt-1); M-phase inducer phosphatase 2
			S22AK_MOUSE_10000	Q80UJ1	
			S22A6_MOUSE_10000	Q8VC69	
			PGFRA_MOUSE_1000	P26618	
			CAH15_MOUSE_10000	Q99N23	
			OPRM_MOUSE_10	P42866	
			HYES_MOUSE_10000	P34914	
			AKT1_MOUSE_10000	P31750	
			MPIP2_MOUSE_10000	P30306	
**13**	4-Hydroxylphenylacetic acid	ZINC00213065	ACE_MOUSE_1000	P09470	Angiotensin-converting enzyme; Solute carrier family 22 member 6; Caspase-1; Glycine receptor subunit α-4; Solute carrier family 22 member 20; RAC-α serine/threonine-protein kinase (Akt-1); Prostaglandin G/H synthase 2; Carbonic anhydrase 15; Hydroxycarboxylic acid receptor 2; Platelet-derived growth factor receptor α(PDGFRα)
			S22A6_MOUSE_10000	Q8VC69	
			CASP1_MOUSE_10	P29452	
			GLRA4_MOUSE_10000	Q61603	
			S22AK_MOUSE_10000	Q80UJ1	
			AKT1_MOUSE_10000	P31750	
			MDR2_MOUSE_10000	None	
			PGH2_MOUSE_1000	Q05769	
			CAH15_MOUSE_10000	Q99N23	
			HCAR2_MOUSE_10000	Q9EP66	
			PGFRA_MOUSE_1000	P26618	

UniProtKB AC means UniProt Knowledgebase number.

### 2.3. Molecular Modeling, Molecular Docking and MD Simulation

**Figure 3:**
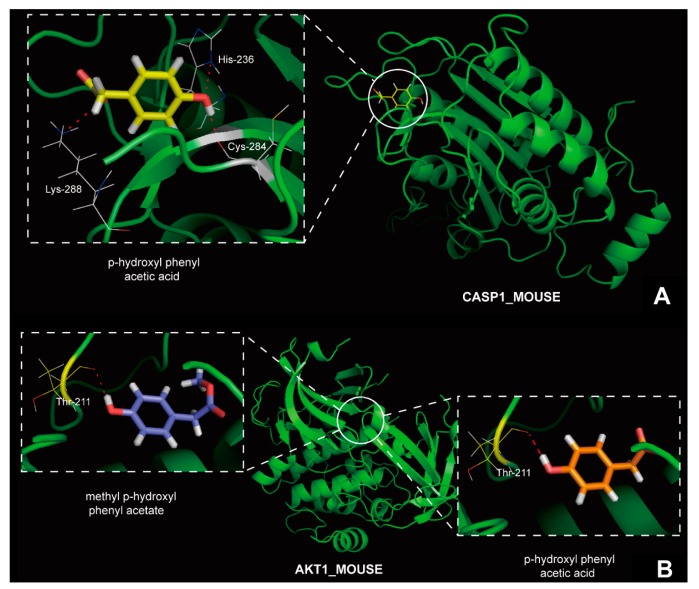
(**A**) The binding mode of compound **13** in caspase-1; and (**B**) the binding mode of compounds **10** and **13** in Akt-1.

From small molecule compounds targeting caspase-1 and Akt-1, compound **13** was selected for virtual screening with molecular modeling, molecular docking and MD simulations, for its effect of hepatic fibrosis. Compound **10** was also selected for targeting Akt-1. Results of molecular docking showed that they are both top-scored for the target proteins, means that they indeed can interact with the targets (Figure 3). MD simulations of the compounds **10** and **13** and their targets were carried out, to further confirm precise binding mechanisms and interaction stability. And the results indicated the stabilities of the dynamics equilibriums for both of them are reliable, and thus provide a reliable basis for further analysis.

### 2.4. The Effect of Compound **10** and Compound **13** on Disease Protection in Mice Hepatocytes

**Figure 4:**
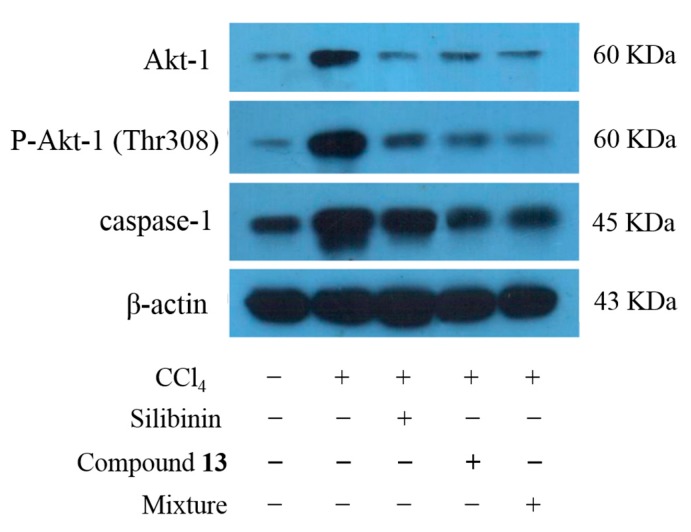
Expression level of caspase-1 and Akt-1 in mice.

Both Akt-1 and caspase-1 were highly expressed when the liver was injured. The expression level of caspase-1 and Akt-1 decreased in the mixture group and compound **13** group compared to the liver injury model group, indicating a hepatic protective effect (Figure 4). From what was discussed above, it can be inferred that compound **13** in *Cichorium intybus* L. reduced the expression level of Akt-1 and caspase-1, thus ameliorated the liver injury to some degree.

## 3. Discussion

In this study, we demonstrated the hepatoprotective effect of *Cichorium intybus* L*.* on mouse model. Furthermore, it can be inferred that *Cichorium intybus* L*.* will have great value in hepatic disease protection, whether for alimentary or medicinal use. Since *Cichorium intybus* L. has complex chemical components, to identify the small molecule compounds and their biological targets has become significant. Therefore, we separated and identified 10 of them. Among them, compounds **2** and **5** were new compounds that had not been reported; compound **6** was isolated from the genus for the first time; and compound **10** was isolated from this plant for the first time. These new compounds may inspire people investigating more about the therapeutic potential of *Cichorium intybus* L*.*, apart from the effective components like Intyboate B that we have already known for its therapeutic effect on hypertension, heart failure, *etc.*

Among the targets of the two selected compounds, Akt-1 and caspase-1 are extremely significant. Akt-1 can prevent apoptosis and high expression level of it may be related to tumorigenesis [8]. Caspase-1 is involved in apoptosis mediated by death receptor [9,10]. The hepatic protective effect of compounds **10** and **13** may be reflected from two aspects: on the one hand, they can down regulate abnormally high expression of Akt-1 and thus prevent the occurrence of tumors; on the other hand, compound **13** also inhibits the expression of caspase-1 and thereby reduces the degree of liver damage via preventing apoptosis. The results of western blot have strongly proved our hypothesis. In addition, since Akt-1 pathway has an extensive biological effect, especially in preventing apoptosis, regulating metabolism and resulting in cytoskeleton reorganization, we can speculate that studies on compounds **10** and **13** are of great significance in the treatment of several diseases including diabetes mellitus.

By applying *in silico* analysis and experimental validation, our study has proven the hepato-protective effect of compounds **10** and **13** in *Cichorium intybus* L. and disclosed its possible molecular mechanisms, which may shed light on designing the drugs aimed at acute liver injury.

## 4. Experimental Section

### 4.1. Experimental Animals and Design

Experiments were conducted on male Kunming mice (Specific pathogen free (SPF) grade), which weighed 20 ± 2 g, and were purchased from the Experimental Animal Center of Xinjiang Medical University (Xinjiang, China), license number: SYXK (Xin) 2003-0001. After 1 week of feeding in a constant temperature of 21–24 °C and 45%–50% relative humidity, the mice were used for experiments. The feedstuffs were provided by the Animal Experimental Center of Shihezi University. Mice were randomly divided into 6 groups of 12 mice each, including the normal control, liver injury model, positive drug silibinin control (200 mg/mL), low-dose drug group (2.0 g/kg chicory), middle-dose group (6.0 g/kg chicory) and high-dose group (18.0 g/kg chicory). The normal group and model group were fed with physiological saline for 7 days, while other groups were fed with the corresponding agents. One hour after the last drug administration, all groups, except the normal control, received a peritoneal injection of 10 mL/kg of 0.2% rapeseed oil solution of CCL_4_. All groups were allowed free access to pure water but no access to food. After 20 h, blood and liver tissue samples were harvested for further examination.

### 4.2. Measurement of Markers of Liver Function and Enzyme Activity

The blood was centrifuged at 3000 r/min at 4 °C for 10 min to separate the serum. Markers of liver function, including aspartate transaminase (AST) and alanine transaminase (ALT), were measured with a UV spectrophotometer (Shimadzu UV-2401, Shimadzu, Kyoto, Japan). The liver tissue was prepared as a 10% homogenate (*w*/*v*). MDA was measured by using a MDA kit, with a UV spectrophotometer (Shimadzu UV-2401) at 532 nm. GSH was measured by using a GSH kit, with a UV spectrophotometer (Shimadzu UV-2401) at 420 nm.

### 4.3. Histopathological Observations in the Liver

Mouse liver tissue was fixed with 10% formalin for 24 h, dehydrated with a sequence of ethanol solutions, and embedded in paraffin. Serial sections were cut at a 5 μm thickness and stained with hematoxylin-eosin (HE) and masson tri-chrome. Stained tissue sections were assessed for the detection of changes in the magnitude of liver injury using a photomicroscope.

### 4.4. Detection of ROS and Immunostaining

Intracellular ROS levels were measured using dichloro-dihydro-fluorescein diacetate (DCFH-DA). For flow cytometric analyses, cells were exposed to 10 μM DCFH-DA (Invitrogen, San Diego, CA, USA) for 5 min. The cells were then analyzed using a FACSCalibur flow cytometer (BD Biosciences, San Jose, CA, USA) using the FL-1 channel (515–545 nm). The mean fluorescence intensity (MFI) was analyzed using CellQuest Pro 4.0.2 software (BD Biosciences, San Jose, CA, USA), and quantification was performed using WinMDI 2.8 software (The Scripps Institute, La Jolla, CA, USA). Small cellular debris was excluded by gating on a forward scatter plot [11,12,13,14,15].

### 4.5. Isolation and Identification of Main Components in Cichorium intybus L.

We isolated 14 compounds and identified 10 of the structures by applying Sephadex LH-20 (General Electric, Fairfield, CA, USA), Open ODS (Octadecylsilyl, Shimadzu, Kyoto, Japan), UPLC-TOF-MS (ultra performance liquid chromatography/time of flight mass spectrometry, Waters, Milford, MA, USA), *etc.* In brief, herbs of *Cichorium intybus* L. (10.0 kg) were extracted with EtOH-H_2_O (95:5, *v*/*v*) three times under conditions of reflux for 3 h each time. The combined EtOH extracts were concentrated, suspended in H_2_O, and then partitioned with CHCl_3_ and EtOAc, to yield CHCl_3_-soluble fraction (46.0 g) and EtOAc-soluble fraction (24.0 g). An aliquot of the CHCl_3_-soluble fraction (40.0 g) was subjected to silica gel CC with a gradient of petroleum ether (PE)/EtOAc to afford fourteen fractions A–N (100:0–100:100). Fraction C (PE-EtOAc, 100:1) (0.0748 g) was applied to silica gel CC eluted with petroleum ether (PE)/EtOAc (100:2) to produce compound **13** (30.0 mg). An aliquot of the CHCl_3_-soluble fraction (20.0 g) was subjected to silica gel CC with a gradient of CHCl_3_/MeOH to afford eight fractions A–H (100:0–100:10). Fraction F (CHCl_3_/MeOH, 100:6) (0.5 g) was applied to silica gel CC and Sephadex LH-20 (General Electric, Fairfield, CA, USA) successively, then separated via HPLC (Shimadzu, Kyoto, Japan), with MeOH-H_2_O (45:55, *v*/*v*) as mobile phase, to produce compound **10** (12.0 mg). The structures of compounds **10** and **13** were identified by comparison of their spectroscopic data with those reported in the literature [16,17,18,19,20]. The relevant chromatograms and mass spectra data of the active compounds are as follows.

Compound **10**: colorless acicular crystal (MeOH); HR-ESI-TOF-MS 167.0705 [M + H]^+^ (calculated 167.0708, C_9_H_11_O_3_); ^1^H-NMR (CD_3_OD, 600 MHz) δ: 7.07 (2H, d, *J* = 8.4 Hz, H-2, H-6), 6.72 (2H, d, *J* = 8.4 Hz, H-3, H-5), 3.66 (3H, s, –OCH_3_), 3.52 (2H, s, H-7); ^13^C-NMR (CD_3_OD, 150 MHz) δ: 173.1 (C-8), 156.2 (C-4), 129.9 (C-2, C-6), 124.5 (C-1), 114.9 (C-3, C-5), 50.9 (C-1ʹ), 39.5 (C-7).

Compound **13**: colorless acicular crystal (MeOH); HR-ESI-TOF-MS 153.0549 [M + H]^+^ (calculated 153.0551, C_8_H_9_O_3_); ^1^H-NMR(CD_3_OD, 600 MHz) δ: 7.00 (2H, d, *J* = 8.4 Hz, H-2, H-6), 6.67 (2H, d, *J* = 8.4 Hz, H-3, H-5), 3.46 (2H, s, H-7), 9.27 (1H, br s, OH); ^13^C-NMR (CD_3_OD, 150 MHz) δ: 176.1 (C-8), 157.4 (C-4), 131.3 (C-2, C-6), 126.8 (C-1), 116.2 (C-3, C-5), 41.1 (C-7).

### 4.6. Predictive Targets of Small Molecule Compounds

Target proteins of the small molecule compounds were obtained from the similarity ensemble approach (SEA) (http://sea.bkslab.org/) [21]. An assistant tool for searching data from SEA dock was developed to simplify this process. The assistant tool can view the source code of the result page and test if the content matches the pattern that we set. If it does match, the program will copy it into a buffer and dump it into a file. Then the functions of target proteins and connections between them were obtained by applying Gene Ontology (GO) (http://www.geneontology.org) and KEGG Orthology (KO) (http://www.genome.jp/kegg/ko.html) [22,23,24]. Furthermore, we selected target proteins involved in disease pathogenesis in liver.

### 4.7. Molecular Modeling and Molecular Docking

We selected 2 effective small molecule compounds and 2 proteins as receptors. Before the docking, we downloaded initial three-dimensional geometric co-ordinates of the X-ray crystal structures of compounds and proteins from the Protein Data Bank (PDB) (http://www.pdb.org/pdb/home/home.do). Molecular docking was performed using the UCSF DOCK6.5 program (University of California, San Francisco, CA, USA), which utilized DOCK algorithm to address rigid body docking by superimposing the ligand on to a negative image of the binding pocket [25,26,27].

### 4.8. MD Simulations of Complexes

Refinements of 3D structures of complexes were done using 10 ns molecular dynamic (MD) simulations. MD simulation of the complex was carried out with the GROMACS 4.5.4 (KTH Royal Institute of Technology, Stockholm, Sweden) [28] package using the GROMOS96 43a1 force field [29]. The lowest binding energy (most negative) docking conformation generated by CDOCKER module embedded in the Accelrys Discovery Studio 3.5 (Accelrys, San Diego, CA, USA) was taken as initial conformation for MD simulation. The topology parameters of proteins were created using the Gromacs program. The topology parameters of ligands were built by the Dundee PRODRG server (University of Dundee, Dundee, UK). The complex was immersed in a cubic box of simple point charge (SPC) water molecules. Eight and eleven sodium counter-ions were added by replacing water molecules to ensure the overall charge neutrality of the receptors simulated system, respectively. To release conflicting contacts, energy minimization was performed using the steepest descent method of 5000 steps followed by the conjugate gradient method for 5000 steps. MD simulation studies consist of equilibration and production phases. To equilibrate the system, the solute was subjected to the position-restrained dynamics simulation (NVT (constant number (N), volume (V), and temperature (T)) and NPT (constant number (N), pressure (P), and temperature (T)) at 300 K for 300 ps. Finally, the full system was subjected to MD production run at 300 K temperature and 1 bar pressure for 5000 ps. For analysis, the atom coordinates were recorded at every 0.5 ps during the MD simulation.

### 4.9. Western Blot Analysis

The expression levels of the selected target proteins (Akt-1 and caspase-1) in liver tissues were measured by applying western blot. Liver tissues were lysed in a buffer containing 1% Triton X-100, 50 mM Tris (pH 7.5), 10 mM EDTA, 0.02% NaN_3_, and a protease inhibitor cocktail (Roche Boehringer Mannheim Diagnostics, Mannheim, Germany). Following one freeze–thaw cycle, the cell lysates were centrifuged at 10,000× *g* at 4 °C for 20 min and boiled in sample buffer for 5 min. The proteins were then subjected to SDS–PAGE (sodium dodecyl sulfate polyacrylamide gel electrophoresis) and transferred to a PVDF (polyvinylidene fluoride) membrane (Millipore, Billerica, MA, USA) using a semi-dry electroblotting system. After blocking with 5% skim milk in PBS, the membranes were incubated with primary antibodies (1:1000 dilution) at 4 °C overnight. The membranes were then washed with PBS containing 0.05% Tween 20 and incubated with horseradish peroxidase (HRP)-conjugated secondary antibodies (1:5000 dilution) at room temperature for 1 h. After washing, the membranes were soaked in ECL solution (PerkinElmer Life Sciences Inc., Boston, MA, USA) for 1 min, according to the manufacturer’s instructions, and exposed to film (BioMax; Eastman Kodak, Rochester, NY, USA). Other materials used for protein analyses were purchased from Sigma-Aldrich (Sigma-Aldrich, St. Louis, MO, USA) [30,31,32].

### 4.10. Statistical Analysis

All the presented data and results were confirmed in at least three independent experiments. The data are expressed as means ± S.D. Statistical comparisons were made by One-way ANOVA and Student’s *t*-test. *p* < 0.05 was considered statistically significant.

## 5. Conclusions

This study demonstrates the hepatic protective effect of *Cichorium intybus* L. and active compounds in *Cichorium intybus* L. The active compounds ameliorate liver injury by acting on Akt-1 and caspase-1, which are related to apoptosis or autophagy. In summary, our results may shed light on the complex molecular mechanisms of *Cichorium intybus* L. acting on hepatocytes and ameliorating liver injury.

## Abbreviations

CIE, *Cichorium intybus* L. extract; AST, aspartate aminotransferase; ALT, alanine aminotransferase; GSH, glutathione; SOD, superoxide dismutase; MDA, malondialdehyde; ROS, reactive oxygen species; Akt-1, RAC-α serine/threonine-protein kinase; CCL_4_, carbon tetrachloride.
